# A 2022 Update on Extensive Stage Small-Cell Lung Cancer (SCLC)

**DOI:** 10.7150/jca.75622

**Published:** 2022-07-18

**Authors:** Bryan Oronsky, Nacer Abrouk, Scott Caroen, Michelle Lybeck, Xiaoning Guo, Xiaohui Wang, Zhongwen Yu, Tony Reid

**Affiliations:** 1 EpicentRx Inc., Department of Clinical Research, 11099 North Torrey Pines Road, Suite 160, La Jolla, CA 92037, USA; 2 SciClone Pharmaceuticals Co., Ltd. Department of Clinical Research, 22 Floor, Shanghai Central Plaza, No. 381 Middle Huaihai Road, Huangpu, Shanghai 200020, China; 3 Clinical Trials Innovations, Mountain View, CA, 94043, USA

**Keywords:** SCLC, platinum chemotherapy, immunotherapy, checkpoint inhibitor therapy

## Abstract

For close to 40 years small-cell lung cancer (SCLC) was adrift, as listless, and as idle as a painted ship on a painted ocean, with nary a breeze to blow in the direction of clinical progress or change. The preferred decades-old first line regimen was etoposide-platinum, to which ≥50% of patients respond, followed by decades-old, tired topotecan in second line for platinum sensitive patients, full stop, because there were no approved therapeutic options (nor generally any compelling experimental ones) in third line or beyond. In 2012 SCLC was designated by the U.S. Congress as a “recalcitrant” tumor type and for good reason: because most patients relapse, after the generally favorable response in first line, respond poorly, if at all to subsequent therapies, and rarely survive beyond 1 year.

A significant sea change occurred in 2018 with the approval of nivolumab followed by pembrolizumab and atezolizumab in 2019, durvalumab in 2020, accelerated approval for lurbinectedin in 2020 and trilaciclib in 2021 for myelosuppression. In 2021, the US indications for nivolumab and pembrolizumab were withdrawn. Suddenly, a tumor type, whose name was virtually synonymous with stalled progress and movement, and which was much less well studied and funded than its more prevalent cousin, non-small cell lung cancer (NSCLC), finds itself in the eye of the storm, that is, at the epicenter of an intense flurry and ferment of activity, not all of it positive. This review surveys approved drugs and select up-and-coming ones in development for extensive stage SCLC.

## Introduction

In the United States, small-cell lung cancer (SCLC) accounts for approximately 15% of all new lung cancer cases^1^ where its incidence is on the decline due to decreased rates of cigarette smoking, which serves as the most established risk factor likely through a DNA damage-related mechanism^2^. By nature, and by predisposition, this highly aggressive neuroendocrine tumor doubles rapidly and disseminates widely early on, so that 80-85% of patients are diagnosed with advanced or extensive disease (ES-SCLC).^3^ According to the tumor, node, metastasis (TNM) staging system from the American Joint Committee on Cancer (AJCC), extensive-stage disease is any T, any N, M1a/b, and T3-4, due to involvement of multiple lung nodules.^4^ By contrast, limited-stage SCLC is defined as any T, any N, M0 except for T3-4, due to multiple lung nodules that do not fit in a single radiation field. Approximately 10-25% of patients are diagnosed with brain metastases at initial presentation, and an additional 40-50% will develop them during their disease.^5^ Limited stage SCLC (LS-SCLC), which has not metastasized is potentially curable, whereas ES-SCLC is not. The cornerstone of treatment management for ES-SCLC since the 1980s is platinum-based chemotherapy, etoposide-platinum (EP), to which the tumors are initially highly sensitive. However, in accordance with the dictates of eco-evolutionary dynamics^6^, intense elimination of sensitive clones inevitably leads to the development of chemoresistance, treatment failure and death. Indeed, overall response rates (ORR) of ≥50% to first line platinum-etoposide are as impressive as they are transient, since median survival time^7^ is around 1 year or less and median progression-free survival in first-line even after the addition of PD-L1 inhibitors barely exceeds 5 months, which contributes to perceptions of futility and nihilism.^8^, ^9^ The response rates to second-line topoisomerase I inhibitor therapy such as topotecan are below 20% overall.^10^ Therein lies the central paradox of SCLC, that it is at once exquisitely chemosensitive and inevitably chemoresistant to whatever is subsequently tried, resulting in significant attrition after each line of therapy (only 50% and 22% receive second-line therapy and third-line therapy, respectively) 11 and an overall static treatment paradigm that reinforces perceptions of therapeutic futility and nihilism.

For 40 years SCLC was consigned to isolated backwater status, underfunded, and understudied due to its recalcitrant aggressiveness and stigmatized as a disease of smokers, only to re-emerge with the landmark approval of nivolumab in 2018, which ushered in a new era of immunotherapy and all at once lifted the curse and the taboo that had attached themselves to SCLC. The premise of immunotherapy in SCLC is that the high tumor mutational burden (TMB) from inveterate tobacco abuse enhances the likelihood of response to checkpoint inhibitors (CIs) 12, which may or not be the case, given how modest the survival improvement with CIs, despite a seemingly low bar. Likewise, for the strong association of SCLC with autoimmune paraneoplastic syndromes like anti-Hu encephalomyelitis 13 and the universal loss of both the RB1 (encoding RB) and TP53 (encoding p53) tumor suppressor genes, which may drive immunogenicity through genetic instability.^14^

A spate of approvals swiftly followed: pembrolizumab and atezolizumab in 2019, durvalumab in 2020 and accelerated approval for lurbinectedin in 2020. In 2021 the US Food and Drug Administration (FDA) approval statuses of nivolumab and pembrolizumab were withdrawn, which winnowed down the field from 8 (platinum doublet, topotecan, nivolumab, pembrolizumab, atezolizumab, durvalumab, lurbinectedin, trilaciclib) to 6. The FDA approval process is based on (1) overall survival (OS), which is, by far, the preferred endpoint and the one most often used, (2) progression-free survival, i.e, the time until cancer recurrence, which is, in general, considered to be less clinically meaningful than OS or (3) response rate (RR), i.e, the percent of patients whose tumors shrink or disappear, which is also considered, in general, with exceptions, to be less clinically meaningful than OS. In this manuscript only the currently approved treatments for ES-SCLC in the United States are briefly surveyed as well as select potentially promising experimental options in development.

## 8^th^ Tumor-Node-Metastasis (TNM) Classification

Typically categorized as limited disease (LD-SCLC) and extensive disease (ED-SCLC) with LD confined to the ipsilateral hemithorax and, thus, theoretically encompassable within a single radiation field, SCLC has also more recently incorporated the new tumor-node-metastasis (TNM) staging system that was developed for non-small cell lung cancer (NSCLC). According to edition 8 of this classification from the American Joint Committee on Cancer (AJCC), limited-stage SCLC is defined as any T (except for T3-T4, which extends beyond a single radiation field), any N and M0. 4 Extensive-stage disease is defined as any T including T3-T4, any N and M1a (pulmonary contralateral metastases or pleural/pericardial effusion) and M1b (extrapulmonary metastases). 15

## Molecular Classification and Potential Therapeutic Sensitivities

**Figure 1:**
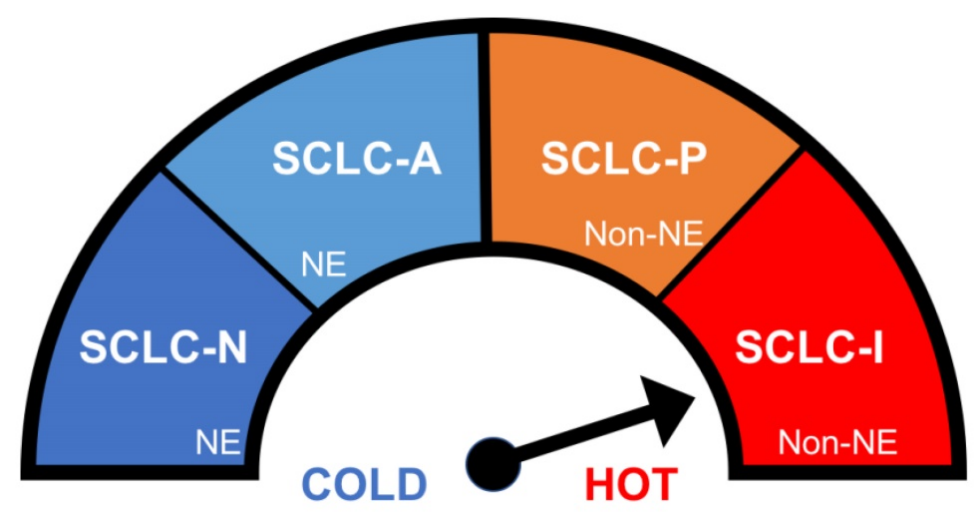
Small cell lung cancer (SCLC) patients can be divided into subtypes, based on transcription factor, with characteristic immunogenic profiles which in turn likely affect treatment responses. The schematic below illustrates the immunogenic spectrum ranging from most 'cold' (SCLC-N) to most 'hot' and potentially most likely to respond to immune checkpoint inhibitor therapy (SCLC-I). Abbreviations: Neuroendocrine (NE), Non-neuroendocrine, (Non-NE),

Although SCLC is classically regarded as a single, monolithic entity, different subgroups with varying therapeutic sensitivities have been identified with immunohistochemistry from murine models and animal tumors based on the differential expression status of key transcription factors 16 as summarized in Figure 1. The “classic” or SCLC-A molecular subtype, as the most common subtype, defined by activation of the transcription factor, ASCL1, which represents around 40-50% of SCLC, is variably sensitive to platinum. The subtype called SCLC-N, defined by the activation of the transcription factor, NEUROD1, which represents around 25-30% of SCLC, is resistant to platinum but possibly sensitive to DNA damage response inhibitors such as poly (ADP-ribose) polymerase (PARP) inhibitors. The non-neuroendocrine subtypes, SCLC-P, defined by the activation of the transcription factor, POU2F3, representing 7-16%, which is the most sensitive to platinum and PARP inhibitors, and SCLC-I, defined by activation of the transcription factor, YAP-1, representing around 15%, which is generally resistant to platinum. Of note, SCLC-I displays an immune-inflamed phenotype (the “I” stands for “inflamed”), possibly suggesting a heightened sensitivity to checkpoint inhibitors.^17^

## First Line

Until recently, before the introduction of checkpoint inhibitors, the standard of care for first‐line treatment of ES‐SCLC was platinum‐based doublet chemotherapy with or without prophylactic cranial irradiation (PCI). Platinum-based chemotherapy involves four to six cycles of cisplatin or carboplatin plus etoposide in Europe and North America, and platinum plus irinotecan in Japan.^18^ Carboplatin is generally used in preference over cisplatin due to its similar efficacy and lower toxicity. Despite its exquisite chemosensitivity, most patients relapse within the first year of treatment: if the relapse occurs during treatment, they are termed “platinum-refractory”, if the relapse occurs within 90 days from the treatment interruption they are termed “platinum-resistant” and if the relapse occurs 90 days or more after treatment they are termed “platinum-sensitive”.^19^ In platinum-sensitive relapse, rechallenge with first-line chemotherapy is preferred over topotecan.^20^ Prior to immunotherapy, the addition of a third agent to this therapeutic backbone was not beneficial.

### a) Immunotherapy

However, the PD-L1 inhibitors, atezolizumab and durvalumab, received FDA approval in March of 2019 and 2020, respectively, in combination with chemotherapy, thereby permanently altering the treatment landscape after decades of stagnation. These approvals were based on practice-changing data from two controlled trials, IMpower133 and CASPIAN, randomized against standard of care chemotherapy. In IMpower133, atezolizumab plus etoposide and carboplatin demonstrated improved overall survival (OS) (hazard ratio [HR], 0.70; 95% confidence interval [CI], 0.54-0.91; *p* = .0069), with a median OS of 12.3 months compared to 10.3 months for etoposide and carboplatin alone. However, only 12.6% of patients remained progression-free at 1 year and the median PFS was 5.2 months for atezolizumab versus 4.3 months for placebo [HR, 0.77; 95% CI, 0.62-0.96; P = .02]. 21 In CASPIAN, durvalumab plus etoposide and either cisplatin or carboplatin also demonstrated improved OS with a hazard ratio of 0.73 (95% CI 0.59-0.91; p=0.0047]); median overall survival was 13.0 months (95% CI 11.5-14.8) in the durvalumab plus platinum-etoposide group versus 10.3 months (9.3-11.2) in the platinum-etoposide group, with 34% (26.9-41.0) versus 25% (18.4-31.6) of patients alive at 18 months. 7 However, the median PFS was 5.1 months for durvalumab vs. 5.4 months for placebo. Also, the addition of tremelimumab, a human IgG2 monoclonal antibody targeting CTLA-4,^22^ to durvalumab plus platinum-etoposide did not significantly improve OS versus platinum-etoposide.^23^

At maintenance, atezolizumab was given every 3 weeks and durvalumab every 4 weeks until disease progression, which is typically how these agents are dosed after 4 cycles of combination therapy.

Another phase 3 randomized trial, KEYNOTE-604, which combined pembrolizumab with platinum etoposide (EP) versus placebo plus EP in the frontline setting for ES-SCLC met its progression-free survival endpoints but missed the OS endpoint (HR, 0.80; 95% CI, 0.64-0.98). 24

Therefore, of the several immunotherapy combinations that have been tried in SCLC, only the anti-PD-L1 inhibitors, atezolizumab and durvalumab, significantly improved survival. It is unknown whether PD-L1 inhibitors are intrinsically superior in SCLC or whether factors such as clinical trial design, site selection, patient variables like age, morbidities etc. or coincidence played a role.

### b) Trilaciclib

In 2021, the CDK 4/6 inhibitor, trilaciclib, received approval as a myeloprotectant not a cytotoxic based on three randomized, double-blind, placebo-controlled Phase 2 studies in ES-SCLC, G1T28-02 (NCT02499770), G1T28-05 (NCT03041311), and G1T28-03 (NCT02514447), which in aggregate randomized 245 patients to receive trilaciclib or a placebo prior to treatment on days 1 through 3 of each 21-day cycle. In a pooled analysis of all three studies, trilaciclib pretreatment reduced the duration and the incidence of severe neutropenia in the first cycle of chemotherapy compared to placebo, although overall survival and progression-free survival were not improved. 25,26,27

In G1T28-02, trilaciclib was given before carboplatin and etoposide in first-line SCLC. In this trial, patients were administered either trilaciclib or placebo on days 1 through 3 of each 21-day cycle.

In G1T28-05, patients received either trilaciclib or placebo on days 1 through 3 of each 21-day treatment cycle for up to 4 cycles as part of the induction phase followed by atezolizumab, given every 21 days, as part of the maintenance phase.

In G1T28-03, patients were given either trilaciclib or placebo prior to topotecan on days 1 through 5 of each 21-day cycle of topotecan in second line ES-SCLC.

The primary myelopreservation end points included duration and incidence of severe neutropenia (DSN), which was defined as grade 4 in cycle 1. Secondary myelopreservation end points included anemia and thrombocytopenia.

Trilaciclib significantly decreased most measures of multilineage chemotherapy-induced myelosuppression and the need for supportive care interventions. The mean DSN with trilaciclib and placebo was 0 compared with 4 days for placebo, respectively (*P* <.0001).

Post cycle 1, 8.9% of the trilaciclib-treated patients required 1 or more dose reductions of chemotherapy versus 30.3% of the placebo-treated patients. Additionally, fewer trilaciclib-treated patients reported serious toxicities (6.5%) or needed treatment with intravenous antibiotics (19.5%) compared with placebo-treated patients (10.1% and 23.5%), respectively.

### c) Tiragolumab

Tiragolumab, an anti-TIGIT antibody checkpoint inhibitor, which is complementary to but distinct from the anti-PD-1/PD-L1 pathway, failed to meet its co-primary endpoints of PFS and OS in a 490-patient randomized Phase 3 trial called SKYSCRAPER-02 with first line atezolizumab and carboplatin plus etoposide versus atezolizumab and carboplatin plus etoposide. Previously, tiragolumab received breakthrough therapy designation from the FDA with atezolizumab in the first-line treatment of patients with metastatic highly expressing PD-L1 NSCLC whose tumors do not harbor any EGFR or ALK aberrations. 28

## Maintenance Immunotherapy after First Line Platinum Doublet

The inevitability of eventual tumor relapse after completion of first-line chemotherapy is the basis for the inclusion of maintenance therapy. However, the results with immunotherapy have been inconsistent at best. In the phase 3 CheckMate-451 trial, patients with responses after completion of first-line chemotherapy were randomized 1:1:1 between nivolumab monotherapy, nivolumab plus ipilimumab, or matching placebo as maintenance therapy. 29 The combination of nivolumab plus ipilimumab did not improve OS, the primary endpoint, over placebo (median 9.2 v 9.6 months) but PFS was slightly better (median 1.7 v 1.4 months).

Only the addition of anti-PD-L1 therapies, atezolizumab and durvalumab, to the standard platinum-etoposide chemotherapy, and then as maintenance, led to improved PFS and OS. 30 In contrast, the use of pembrolizumab, an anti-PD1 therapy, in combination with platinum-etoposide and then as maintenance significantly improved PFS but not OS. 31

Unlike in NSCLC, useful biomarkers for the *a priori* selection of SCLC patients that are predicted to benefit from immunotherapy are lacking especially since most SCLC tumors lack PD-L1 expression. 32,33

## Second Line

Following relapse, patients are categorized as “sensitive” and “resistant” according to duration of response to initial chemotherapy. Relapse > 90 days after the last exposure to first-line chemotherapy is termed sensitive provided a complete or partial response was achieved, whereas failure to respond to the first-line chemotherapy or recurrence < 90 days after the last exposure to first-line chemotherapy is termed “resistant”. Second-line options include rechallenge with a first-line platinum regimen, which is usually reserved for chemosensitive disease, topotecan or lurbinectedin.

### a) Topotecan

Topotecan, a topoisomerase 1 inhibitor, was approved in 1998 based on a randomized Phase 3 trial clinical trial with 211 patients that relapsed at least 60 days after their initial treatment. Patients received either an intravenous infusion of topotecan (1.5 mg/m²) as a single agent for 5 consecutive days every 3 weeks or CAV (cyclophosphamide, 1,000 mg/m², Adriamycin, 45 mg/m², and vincristine, 2 mg) administered intravenously on day 1 every 3 weeks. While median survival, time to progression and overall response rate were comparable between the two groups, toxicity was not: shortness of breath (P = .002), fatigue (P = .032), hoarseness (P = .043), and anorexia (P = .042) were significantly improved in favor of topotecan as was interference with daily activities (p = 0.023). 34

Eckardt *et al.* conducted a randomized phase 3 trial to compare PO topotecan with IV topotecan in relapsed SCLC, in which, out of 309 patients, 153 patients received oral topotecan 2.3 mg/m^2^ daily for five consecutive days every three weeks and 151 received IV topotecan 1.5 mg/m^2^ daily for five consecutive days every three weeks. The study showed a similar ORR in both arms of 18.3% *vs.* 21.9% respectively, and no difference in median time to response (6.1 weeks for both), median duration of response (18.3 weeks *vs.* 25.4 weeks), and median time to progression (11.9 weeks *vs.* 14.6 weeks).^35^

Nevertheless, topotecan is associated with dose-limiting hematologic toxicities such as neutropenia, anemia and thrombocytopenia, non-hematologic toxicities such as fatigue, alopecia, nausea, and diarrhea and a response rate <20%, which limits its use in practice. Also, topotecan is approved only for those patients with platinum sensitivity.

### b) Carboplatin plus etoposide rechallenge

i. In an open-label, 1:1 randomized, phase 3 French trial from 2020 with progression free survival (PFS) as the primary endpoint, 164 patients with histologically or cytologically confirmed advanced stage IV or locally relapsed small-cell lung cancer, who responded to first-line platinum plus etoposide treatment but relapsed or progressed ≥90 days after completion of first-line treatment were enrolled.

The results demonstrated a median progression-free survival that was significantly longer in the platinum doublet group than in the topotecan group (4.7 months vs 2.7 months, hazard ratio 0.57; p=0.0041), suggesting that carboplatin + etoposide is the preferred option in patients with sensitive relapsed SCLC. 36

ii. In an open-label, 1:1 randomized, phase 3 180 patient Japanese trial from 2016 with overall survival (OS) as the primary endpoint, cisplatin + etoposide + irinotecan was compared with topotecan. Overall survival was significantly longer in the combination chemotherapy group (median 18.2 months vs. 12.5 months), leading to the authors to suggest replacement of topotecan as second line standard of care with the combination of cisplatin + etoposide + irinotecan. 37

### c) Lurbinectedin

A synthetic derivative of the marine drug trabectedin, lurbinectedin 38 is a DNA binding agent that selectively inhibits RNA polymerase II transcription, and which has demonstrated activity against SCLC both as a single agent and in combination with doxorubicin.

Based on a single arm, multicenter, phase 2 basket trial in a mixed population of platinum-sensitive and platinum-resistant patients treated with 3.2 mg/m^2^ of IV lurbinectedin every 3 weeks, which demonstrated an ORR of 35.2% (95%CI: 26.2-45.2), an overall median duration of response of 5.3 months (95%CI: 4.1-6.4), a median progression free survival (PFS) of 3.5 months (95%CI: 2.6-4.3) and a median OS of 9.3 months (95%CI: 6.3-11.8), lurbinectedin received accelerated FDA approval on June 15, 2020 in second line after progression on platinum-based chemotherapy. 39

Hematologic toxicities included Grade 4 neutropenia in 25% of the patients, Grade 4 thrombocytopenia in 4% and 10% of the patients experienced serious adverse events, of which neutropenia and febrile neutropenia were the most common. Non-hematologic toxicities included fatigue, decreased appetite, and gastrointestinal symptoms.

A phase 3 clinical trial in second line SCLC called ATLANTIS, which compared lurbinectedin plus doxorubicin to physician's choice of either topotecan or CAV, reportedly failed to meet its prespecified OS endpoint. 40 Since ATLANTIS was specified as a confirmatory trial, it is unclear whether, like nivolumab and pembrolizumab, which were also granted accelerated approval but subsequently failed to demonstrate efficacy, this will result in the withdrawal of the approval status of lurbinectedin. In the meantime, lurbinectedin is reportedly under investigation with other checkpoint inhibitors and irinotecan and approval will likely depend on the outcomes of these and other follow-on trials. 41

## Third Line

As mentioned, nivolumab and pembrolizumab having been previously approved for SCLC based on the phase 1/2 CheckMate 032 trial, phase 1b KEYNOTE-028 trial, and phase 2 KEYNOTE-158 trial were withdrawn in 2021, leaving no current third line options.

## Potential Future Options

### a) RRx-001 plus EP (Phase 3)

An antagonist of CD-47, C-MYC 42 and VEGF 43 as well as a reactivator of silenced p53 44 that is associated with both tumor cytotoxicity and normal tissue protection including protection from platinum-based myelotoxicity 45, RRx-001 is in a Phase 3 trial called REPLATINUM (NCT03699956) as a combined cytotoxic/myeloprotectant in third line and beyond SCLC.^46^

### b) Velaparib

Velaparib is a PARP inhibitor, which prevents DNA repair. The PARP enzyme is highly expressed in SCLC.^47^ A randomized Phase 2 study of veliparib plus EP in treatment naïve SCLC patients demonstrated a slight improvement in PFS from 5.6 to 5.8 months without a corresponding benefit in overall survival.^48^ More encouraging was the identification of Schlafen 11 (SLFN11), an RNA/DNA helicase that serves as a potential predictive biomarker for sensitivity to PARP inhibition, since SLFN11 expression, which is high in SCLC, decreases significantly after treatment with veliparib.^49^

### c) Temozolomide

Temozolomide (TMZ) is an oral alkylating agent 50, previously shown to have single agent activity in SCLC, which is known to synergize with PARP inhibitors that prevent repair of temozolomide-induced DNA damage. 51

Pietanza et al. conducted a randomized, double-blind, placebo-controlled study of veliparib (40 mg twice daily, days 1 to 7) or placebo and TMZ (150-200 mg/m^2^/day, days 1 to 5) on a 28-day cycle. 52 The primary endpoint of the study, 4-month PFS, was not reached with no significant differences observed between TMZ/veliparib (36%) and TMZ/placebo (27%). Median PFS and OS was also similar between the two arms. However, PFS and OS were significantly improved in SLFN11-positive tumors treated with TMZ/veliparib (5.7 vs. 3.6 months, (p = 0.009) for PFS) and (12.2 vs. 7.5 months; p = 0.014) for OS).

Additional ongoing studies include the combination of PARP inhibitors with agents that induce DNA damage such as pegylated SN-38, the active metabolite of irinotecan, an inhibitor of topoisomerase I activity (NCT04209595). 53

### d) Antiangiogenics

Given the importance of neoangiogenesis in SCLC and the correlation between decreased survival and a higher serum concentration of VEGF, the combination of immunotherapy and antiangiogenic agents is under investigation on the premise that the vascular normalization, which may result from antiangiogenesis, will lead to better T-cell infiltration in tumors and less hypoxia and will, therefore, synergize with checkpoint inhibitors. An example is AK112, a bispecific antibody against PD-1 and VEGF, that is in a Phase 1b/2a clinical trial (NCT05116007) with carboplatin and etoposide. 54

### e) Other chemoimmunotherapy combinations

New chemoimmunotherapy combinations include 177Lu-DOTATATE, a somatostatin receptor-targeted radionuclide therapy, since SCLC is a neuroendocrine tumor, which expresses somatostatin receptors 55; BMS-986012, an anti-fucosyl-GM1 monoclonal antibody in a Phase 2 trial since fucosyl-GM1, a tumor-associated antigen, is highly expressed on SCLC cells but not on normal tissue; and LB-100, a small molecule inhibitor of protein phosphatase 2A (PP2A), which is overexpressed in SCLC. 56

## Discussion/Conclusion

Small-cell lung cancer is small in terms of incidence, accounting for about 15% of all new lung cancer diagnoses but disproportionately large in terms of its aggressive lethality, which makes it a disease of significant unmet need. Tobacco is the primary cause with the duration and intensity of smoking having a significant effect on relative risk. 57 In contrast with NSCLC, where therapies that target oncogenic drivers such as EGFR or ALK are targetable, hallmark SCLC loss-of-function mutations in the tumor suppressors, tumor protein p53 (TP53) and retinoblastoma 1 (RB1), are, unfortunately, not. 58

Nevertheless, from its once underfunded, underrepresented, and understudied status, SCLC made a comeback in 2018-2019 with the conditional approvals of nivolumab and pembrolizumab, watershed events, given that they reversed the long legacy of failure and frustration attendant on the disease and reframed many taken-for-granted assumptions about the futility of treatment.

Moreover, these events not only revived the previously moribund field as a whole and set the stage for subsequent approvals but also served as a catalyst to evaluate several promising drug candidates with the potential to further revolutionize the treatment landscape. Thus, initially hailed and hyped as breakthroughs, nivolumab and pembrolizumab may have lost the battle having ultimately failed to meet their endpoints in confirmatory trials but they won the war because in their place other checkpoint inhibitors like atezolizumab and durvalumab assumed primacy and established a firm foothold on a previously very slippery SCLC slope with full approvals in first line. From this lofty, established perch, atezolizumab and durvalumab and, by extension, immunotherapy, are potentially combinable in first and later lines with a series of immunotherapies, alkylating agents, (since EP may not be optimized for CIs), CAR-Ts, oncolytic viruses, vaccines, radiotherapy, and other agents or modalities. The high mutational burden of SCLC, which is second only to melanoma, as well as its association with paraneoplastic syndromes, such as Lambert-Eaton, which occurs when the immune system cross reacts with normal and neoplastic tissues, strongly suggests that the tumor should be responsive to immunotherapy strategies. 59

Other promising agents include the small molecule, RRx-001, which, because it inhibits VEGF, activates p53 signaling and downregulates CD47 and c-myc is hypothesized to synergize with first line platinum-based chemotherapy. Likewise, temozolomide (TMZ) may synergize with PARP inhibitors especially in SLFN11-positive tumors since TMZ increases DNA damage, and PARP inhibitors diminish the ability of PARP enzymes to repair DNA damage. Also, the antiangiogenics may increase the effectiveness of immunotherapy through improved local perfusion, and decreased hypoxia.

**Figure 2:**
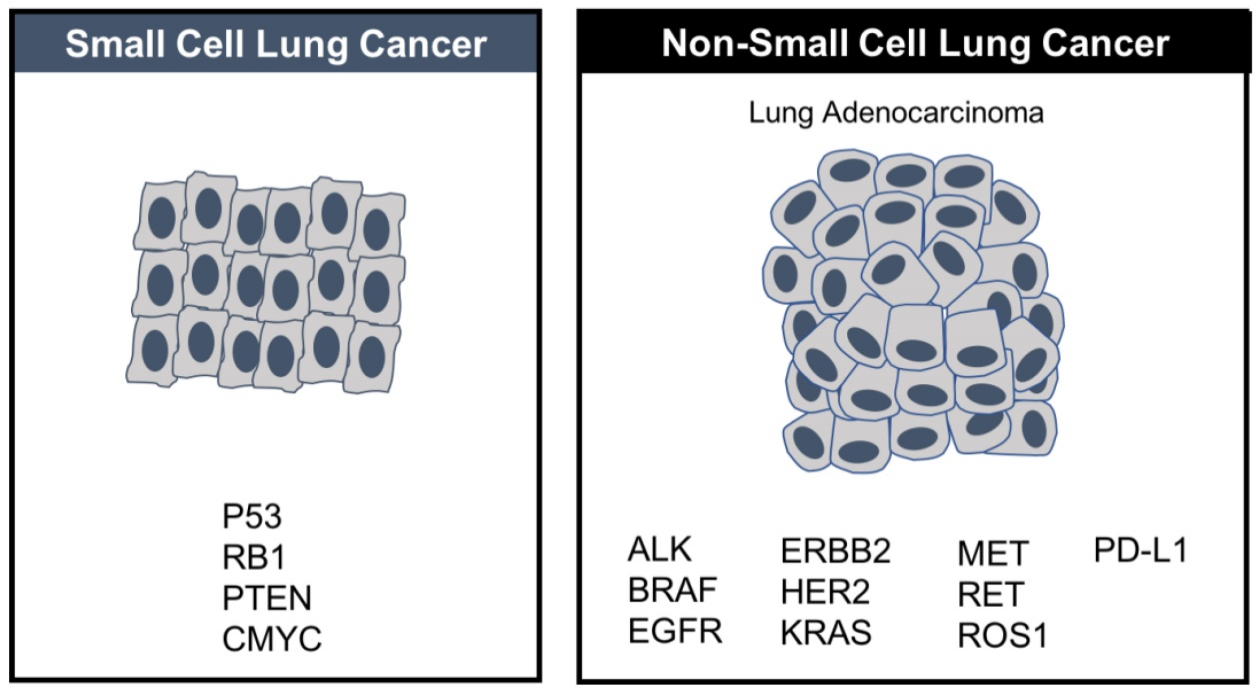
In contrast to NSCLC, where several targeted therapies have been approved based on driver mutations, no targeted therapies have been approved in SCLC, where driver mutations are fewer.

These potentially promising options aside, SCLC is still a very poorly understood, shapeshifting disease that has benefited to a much lesser extent than other tumor types such as NSCLC. In the absence of essential knowledge about the main molecular mechanisms, which underlie escape from immune surveillance, tumor progression and chemoresistance, it is difficult to “crack the code” of SCLC and to design rational treatment strategies for it. The major causes of this knowledge gap are 1) the small sample size of SCLC relative to NSCLC, which makes it more challenging to test new treatments in randomized clinical trials and 2) the paucity of adequate tumor specimens to guide rational drug design as surgery is rarely used to treat extensive stage SCLC 60, which at diagnosis is almost always disseminated. Also, biopsy in the setting of disease relapse is a major challenge, although liquid biopsies enriched from a standard peripheral blood draw may offer a repeatable alternative since circulating tumor cells are abundant in SCLC. Unlike NSCLC, where multiple driver mutations have been identified, few driver mutations exist in SCLC, which is instead almost uniformly linked to loss-of-function alterations in the tumor suppressor genes TP53, PTEN and RB1. 61 (Figure 2)

Biomarkers may offer a way forward with the personalized matching of therapy to baseline tumor subtype, for example, the use of immunotherapy in the I subtype and the use of PARP inhibitors in the P subtypes or in tumors with high SFLN-11 expression so that an individualized paradigm of treatment, considering the tremendous heterogeneity in SCLC, replaces the current monolithic, homogeneous one. Also, while the expression of PD-L1, which is generally low in SCLC and not associated with clinical efficacy, unlike in NSCLC, tumor mutation burden (TMB) appears to be more promising with benefit reported for both single-agent nivolumab and the combination of nivolumab and ipilimumab. 62

Currently extensive stage SCLC is treated according to a general, one-size-fits-all paradigm, which may hopefully soon, with new therapies and biomarkers on the horizon, switch to a more precision-based and personalized one.
